# El islam ante los tratamientos de reproducción asistida: un estudio empírico sobre la relación entre ciencia y religión en Tánger y Barcelona

**DOI:** 10.18294/sc.2023.4492

**Published:** 2023-09-22

**Authors:** Rosa Martínez-Cuadros

**Affiliations:** 1 Doctora en Sociología. Investigadora postdoctoral, Universitat Autònoma de Barcelona. Barcelona, España. rosamartinezcuadros@gmail.com Universitat Autónoma de Barcelona Universitat Autònoma de Barcelona Barcelona Spain rosamartinezcuadros@gmail.com

**Keywords:** Islam, Ciencia, Religión, Técnicas Reproductivas, Bioética, Islam, Science, Religion, Reproduction Techniques, Bioethics

## Abstract

En las últimas décadas hubo un desarrollo significativo de técnicas de reproducción asistida que ayudaron a parejas con dificultades a tener hijos. Estas técnicas han sido bien recibidas en diferentes partes del mundo, y los países musulmanes no han sido una excepción. Desde la perspectiva teórica de la socióloga Michèle Lamont basada en las fronteras o “boundaries”, en el año 2022 se realizaron entrevistas semiestructuradas a 20 profesionales de la salud y líderes de asociaciones musulmanes de Tánger y Barcelona, con el objetivo de analizar, en primer lugar, cómo actores claves musulmanes conciben la relación entre islam y ciencia y; en segundo lugar, cómo se negocia esta comprensión en el caso de los tratamientos de reproducción asistida. Se concluye sobre la complejidad de la delimitación de fronteras en las definiciones de ciencia y religión, y se destaca la importancia de centrarse en casos empíricos para comprender mejor la compleja relación entre los dos ámbitos y entender los debates bioéticos existentes.

## Abstract (en)

In recent decades there have been significant developments in assisted reproduction techniques, which have aided couples with difficulties in having children. These techniques have been well received in different parts of the world, and Muslim countries have been no exception. Adopting sociologist Michèle Lamont’s theoretical perspective on “boundaries”, semi-structured interviews were conducted with 20 health professionals and Islamic community leaders in the cities of Tangier and Barcelona during 2022. The aim of this article is twofold: first, to analyze how key actors in the Muslim community conceive of the relationship between Islam and science; second, to explain how this understanding is negotiated in relation to assisted reproduction treatments. The article concludes with a consideration of the complexities related to drawing the line between the positions of science and religion, and emphasized the importance of examining empirical cases in order to better capture the complex relationship between these two spheres and gain a deeper understanding of existing bioethical debates.

## INTRODUCCIÓN

¿Qué sucede cuando una pareja musulmana no puede tener hijos de forma natural? ¿Qué debates bioéticos emergen sobre el uso de técnicas de reproducción asistida en el islam? En las últimas décadas hubo un desarrollo significativo de técnicas de reproducción asistida que ayudaron a parejas con dificultades para tener hijos. Estas técnicas han sido bien recibidas en diferentes partes del mundo, y los países musulmanes no han sido una excepción. A pesar de que desde el mundo occidental se suele concebir el mundo islámico como retrasado o anticientífico, la apuesta por el conocimiento científico también ha estado presente en contextos musulmanes. Sin embargo, como sucede en otros casos y contextos con la interrelación entre ciencia y religión, los avances científicos han generado debates bióticos sobre sus implicaciones en los principios normativos del islam y han supuesto la consecuente emergencia de lo que podríamos denominar “bioética islámica”^1^^,^^2^^,^^3^. Esto tiene una relevancia especial en el caso de las técnicas de reproducción asistida, ya que tiene un impacto directo en dos niveles: a nivel individual, en la toma de decisiones sobre temas personales que afectan la vida cotidiana como la familia y que a veces se dejan en el ámbito privado y, a nivel colectivo, cuando desde los actores y la administración públicos se intenta regular sobre las prácticas y técnicas permitidas. 

Así, a partir de estas premisas y considerando el caso de las técnicas de reproducción asistida como un ámbito privilegiado para analizar la aplicación práctica de la bioética islámica, el objetivo de este artículo es doble: en primer lugar, analizar cómo actores claves musulmanes conciben la relación entre islam y ciencia y; en segundo lugar, explicar cómo se negocia esta comprensión en el caso de los tratamientos de reproducción asistida. El artículo, además, incluye el estudio de dos contextos sociales y políticos diferentes. Por un lado, incluye la ciudad de Tánger como una ciudad del sur global y de un país de mayoría musulmana en el que los discursos bioéticos pueden ser moldeados y negociados a través de la ley islámica existente, así como de su contexto político y social. Por otro, analiza Barcelona como una ciudad con un emergente crecimiento de diversidad religiosa y en el que la presencia de personas musulmanas todavía se caracteriza por ser minoritaria. A partir de una perspectiva comparativa entre dos contextos sociales y políticos diferentes, se analizan las implicaciones que tienen estos debates en la salud individual y colectiva de una sociedad que se caracteriza por ser cada vez más compleja y diversa, y con múltiples conexiones migratorias y transnacionales. 

### Religión y ciencia: armonía, conflicto y complejidad

En los últimos años, varios investigadores e investigadoras han propuesto diferentes explicaciones sobre la relación entre religión y ciencia, que van desde la tesis del conflicto hasta los “magisterios no superpuestos”^4^^,^^5^^,^^6^. Siendo “religión” y “ciencia” categorías que también aparecen en el debate público, enfoques recientes también han señalado la importancia de ir más allá de estas categorías simplistas y centrarse en la compleja interacción entre la ciencia y la creencia^7^. Entonces, al investigar estos temas, se puede promover fácilmente la idea de que existe un conflicto entre la ciencia y la religión, a pesar de que no sea intencional^7^. Para algunos autores, esto también explica en parte su preferencia por utilizar el concepto de “creencia” o *belief* e incluir un enfoque no religioso. Según Gülker^6^, otra forma de superar la tesis del conflicto es analizar la relación entre religión y ciencia de manera empírica. Por lo tanto, acercándose a las diferencias empíricas y analizando los límites entre ellas, podemos mostrar que son categorías controvertidas.

En el caso concreto de la relación entre el islam y la ciencia, el análisis puede volverse aún más complejo. Como ya han afirmado algunos autores, existe un discurso general sobre el hecho de que el islam no permite a los musulmanes llegar al momento de iluminación que sí proporciona la ciencia^8^. Es decir, sobre todo desde lo que consideramos “mundo occidental”, es común asumir que el mundo islámico es atrasado o anticientífico. Este discurso se utiliza para tratar una supuesta evidencia de la incompatibilidad del islam con la “civilización occidental”. Además, incluso portavoces de la ciencia han retratado al islam como opuesto a la ciencia^7^. En esa línea, Jones, Catto, Kaden y Eldson-Baker^7^ analizaron los relatos de los no-musulmanes sobre el islam y la ciencia y mostraron cómo sus descripciones estaban influenciadas por discursos antiislámicos y narrativas sobre la superioridad cultural occidental. Desde la sociología se han focalizado menos en explorar cómo los mismos musulmanes abordan las cuestiones relacionadas con el islam y la ciencia y cómo negocian su significado^8^.

En este contexto, es relevante analizar cómo las propias personas musulmanas negocian las definiciones de ciencia y religión y establecen límites sociales y simbólicos para definir y relacionar islam y ciencia^9^. Por lo tanto, a partir de la aproximación teórica de Michèle Lamont y su perspectiva de “boundaries” o fronteras^9^^,^^10^, esta investigación se basa en el trabajo de definición de límites y en el análisis empírico de las diferencias entre religión y ciencia. Sin embargo, este artículo no se centra exclusivamente en analizar la relación entre estas dos categorías a nivel teórico, sino que también incluye los tratamientos de reproducción asistida como un caso empírico específico en el que los límites entre la ciencia y el islam podrían ser renegociados y en los que emergen debates bioéticos concretos. Por lo tanto, analizo cómo las categorías de “religión” y “ciencia” también pueden ser “categorías de práctica”^11^ que las propias personas musulmanas pueden usar para definir su identidad religiosa y su comprensión de la ciencia. Además, centrarse en el caso empírico de la infertilidad y los tratamientos de reproducción asistida es pertinente ya que es un problema que puede afectar la vida cotidiana de las parejas y las familias musulmanas, además de evidenciar la complejidad de los debates bioéticos en el mundo contemporáneo. Otras investigaciones ya han destacado cómo la relación entre bioética, salud y religión es determinante en la toma de decisiones sobre tratamientos médicos en el mundo contemporáneo^12^^,^^13^, sobre todo para las mujeres^13^, y este estudio pretende ser una contribución a esta línea de literatura. 

### Tratamientos de reproducción asistida y el islam

La infertilidad se define como “la incapacidad de una pareja para concebir después de un año de relaciones sexuales regulares y sin protección”^14^. Esta situación afecta tanto a hombres como a mujeres, especialmente en las sociedades islámicas donde las principales fuentes del islam, el Corán y los Hadices, afirman la importancia del matrimonio y la familia. En 1978, nació el primer bebé probeta con éxito y, desde entonces, se han desarrollado diferentes tecnologías de reproducción asistida. En los países musulmanes, el desarrollo de estas técnicas ha planteado algunos desafíos entre el uso de tecnologías genéticas y el respeto a las leyes religiosas^15^. Los tratamientos de infertilidad son variados y pueden incluir desde la fertilización in vitro hasta la donación de terceros (óvulos y espermatozoides) y la gestación subrogada. 

Como la mayoría de las investigaciones indican, una de las primeras particularidades de estos tratamientos en las sociedades islámicas es que existe una diferencia entre los musulmanes sunníes y chiíes, las dos principales ramas del islam^16^^,^^17^^,^^18^^,^^19^^,^^20^. Mientras que el islam sunní prohíbe las tecnologías que utilizan donaciones de terceros, algunos chiíes aceptan esta posibilidad. Esto también explica por qué la mayoría de las investigaciones sobre la negociación de tratamientos de infertilidad e islam se están llevando a cabo en Irán, un país donde la mayoría de la población musulmana es chií. Además, Irán es el único país musulmán en el que las técnicas de reproducción asistida que utilizan óvulos y espermatozoides de donantes han sido aceptadas por la ley y las autoridades religiosas^18^. Menos investigaciones sobre técnicas de reproducción asistida se han centrado en otros países musulmanes y en la población sunní, a pesar de que este grupo representa aproximadamente entre el 80% y el 90% de todos los musulmanes a nivel mundial. Y menos aún se centran en países no musulmanes o incluso en países europeos en los que la mayoría de los musulmanes son sunníes^21^.

Esta diferencia entre musulmanes sunníes y chiíes evidencia cómo una de las principales cuestiones que marcan la bioética islámica es la importancia del matrimonio y del parentesco. Los discursos bioéticos islámicos parten de una tradición islámica normativa^2^, en la que el matrimonio y el parentesco son relevantes. En ese sentido, Inhorn y Tremyane^1^ hablan de la emergencia de una “*bioethical aftermath*” o “consecuencias bioéticas” para hacer referencia a las consecuencias imprevistas y los nuevos retos éticos y sociales ante los avances de las técnicas de reproducción asistida, especialmente ante el posible anonimato de los donantes. Mientras que el islam no niega los avances científicos ni se opone a ellos per se, los países musulmanes han tenido que regular las técnicas utilizadas para que no choquen con los principios bioéticos islámicos. Una de las preocupaciones claves para los musulmanes sunníes es la importancia de preservar el *nasab*, es decir, los orígenes genealógicos, y asegurar los derechos de los niños de conocer su parentesco. Además, otra preocupación es la posibilidad de incesto entre descendientes de donantes anónimos. Es decir, la imposibilidad de controlar que medio hermanos se puedan enamorar y casar. Por todas estas cuestiones, el uso de donantes está generalmente prohibido en el mundo musulmán sunnita -actualmente mayoritario- considerando la donación como equiparable al adulterio o *zina*^1^.

En el ámbito académico, la generalización de estudios sobre de los musulmanes chiíes puede generar la impresión de que las técnicas de reproducción asistida se utilizan solo en el islam chií y nunca entre los musulmanes sunníes. Sin embargo, en los países musulmanes sunníes se han aceptado los tratamientos de reproducción asistida y se han regulado en concordancia con las moralidades religiosas. Además, otros estudios también han mostrado cómo algunos musulmanes sunníes transgreden la autoridad religiosa y utilizan óvulos de donantes de técnicas de reproducción asistida^18^. En esta línea, resulta importante analizar hasta qué punto la prohibición reproductiva también puede producir “resistencia reproductiva”^19^ contra las prohibiciones legales de un país o las establecidas por los organismos de jurisprudencia islámica. Así, una posible consecuencia es lo que comúnmente se conoce como “turismo reproductivo”, en el que parejas musulmanas migrantes viajan a otros países, especialmente a países de mayoría musulmana, para acceder a tratamientos de fertilidad que sigan la ley islámica^22^. 

A pesar de no ser un área de investigación ampliamente desarrollada, la mayoría de los estudios existentes se centran en las perspectivas religiosas y la opinión pública sobre técnicas de reproducción asistida^14^. Sin embargo, se ha prestado menos atención a las negociaciones que deben llevar a cabo los profesionales de la salud e incluso las parejas infértiles. Teniendo en cuenta que las mujeres pueden sufrir más adversidades y también pueden sufrir violencia doméstica, estigmatización y ostracismo, se debe desarrollar más investigación para comprender cómo las mujeres negocian, gestionan y deciden sus actitudes y prácticas sobre estos tratamientos^23^^,^^24^^,^^25^. Además, algunos estudios ya han señalado que hombres y mujeres pueden tener opiniones diferentes sobre técnicas de reproducción asistida. Por ejemplo, las mujeres podrían estar más abiertas a usar donaciones de terceros como último recurso. Estas tecnologías también tienen una diferencia en cuanto a género: la donación de óvulos y la gestación subrogada parecen ser más aceptadas que la donación de esperma^16^. Las ideas de las parejas infértiles podrían diferir del sistema y los requisitos de los profesionales de la salud^22^. En este sentido, el objetivo de este texto es identificar tensiones y temas claves entre profesionales de la salud y lideres musulmanes, que pudieran servir para una investigación más extensa con mujeres, parejas infértiles o familias que han hecho uso de técnicas de reproducción asistida.

## METODOLOGÍA

Este artículo se basa en una investigación realizada en 2022 como parte de un estudio piloto titulado “*Islam, science and gender: Negotiating boundaries in two case studies in Spain and Morocco*” (SEED 01.106), financiado por la International Research Network for the Study of Science and Belief in Society (INSBS). El objetivo fue explorar los procesos contemporáneos de negociación de los discursos científicos y religiosos entre la población musulmana en dos ciudades diferentes. 

Se realizaron entrevistas semiestructuradas y observaciones etnográficas en dos ciudades: Tánger y Barcelona. Al ser un proyecto exploratorio, decidí centrar la investigación en dos perfiles concretos de actores claves: profesionales de la salud y líderes de asociaciones musulmanes. Debido a la especificidad del tema, y considerando que se trataba de cuestiones íntimas y personales, escogí un perfil de personas que pudieran tener un conocimiento o experiencia previa de la temática analizada. Por un lado, los profesionales sanitarios se incluyeron para poder identificar posibles tensiones y dilemas que ellos vivan en primera persona o a través de sus pacientes. Concebí como “líderes de asociaciones” a responsables de centros religiosos o asociaciones musulmanas. Las entrevistas a este perfil respondían a la voluntad de conocer hasta qué punto estos temas, así como la relación entre ciencia y religión, eran tratados en espacios de socialización y encuentro de personas musulmanas. Adapté un guion de entrevista para cada uno de los perfiles, siempre priorizando que pudieran surgir temas inicialmente no previstos e identificados como relevantes por los informantes. 

Realicé un total de 20 entrevistas, 10 en cada ciudad, a profesionales de la salud y líderes musulmanes, teniendo en cuenta la paridad de género. En Barcelona, debido a la complejidad de encontrar profesionales de la salud que sean musulmanes, contacté con una variedad de especialidades (2 médicos, 2 enfermeras y 1 farmacéutico). También entrevisté a líderes de asociaciones o personas responsables de centros religiosos y asociaciones musulmanas (1 imán, 2 presidentes de asociaciones musulmanas y 2 líderes de asociaciones de mujeres). Los principales informantes los identifiqué a partir de mi experiencia previa realizando proyectos de investigación sobre estas temáticas parecidas en la ciudad. Contacté a los participantes a través de contactos personales de proyectos de investigación anteriores en la ciudad y mediante el método de “bola de nieve”. 

Para realizar las entrevistas en la ciudad de Tánger, realicé una breve estancia en agosto de 2022 y después volví en octubre del mismo año para una estancia de dos semanas. Contacté a profesionales de la salud y asociaciones a través de Internet, buscando en sitios web de “médicos” y perfiles de redes sociales e intenté acercarme a diversos perfiles de profesionales de la salud (1 médico, 2 embriólogos, 1 ginecólogo y 1 farmacéutico). También contacté a líderes religiosos y miembros de asociaciones locales a través de búsquedas en Internet y contactos personales (1 *sheik* o sabio del islam, 2 miembros de asociaciones de mujeres y 2 líderes de asociaciones sociales). Todas las personas informantes eran musulmanes sunníes, a excepción de una chica en Barcelona que formaba parte de una comunidad chií. La investigación en Barcelona se realizó en español o catalán, siguiendo la preferencia de los informantes, mientras que las entrevistas en Tánger se realizaron en inglés, español o francés. Al contar con informantes con estudios universitarios, el idioma no fue un impedimento a la hora de poder contactar con posibles personas entrevistadas. Sin embargo, desde el principio tuve que plantear esta condición al no tener conocimientos de árabe suficientes para tener una conversación. Analicé todas las entrevistas en el idioma en que se desarrollaron, pero para este artículo las he traducido para facilitar su comprensión. Antes de todas las entrevistas ofrecí información detallada del proyecto y pedí consentimiento informado oral, en el que los entrevistados aceptaban ser grabados y eran informados de que los datos obtenidos y publicados serían anonimizados. 

Inicialmente, como parte de la investigación, también planeé la posibilidad de realizar observaciones etnográficas en eventos en mezquitas y asociaciones musulmanas en los que se tratase el tema de la religión y la ciencia. Cuando estaba diseñando el proyecto, identifiqué una mezquita en Barcelona que organizó un evento titulado “Ciencia moderna y salud”. Pensando en la posibilidad de que este tipo de eventos se pudieran repetir, planeé asistir a eventos similares para analizar la información proporcionada, el papel de estos eventos e identificar posibles informantes para entrevistar. Sin embargo, durante el período del proyecto, solo pude observar un segundo evento y no se organizaron más eventos similares ni en Barcelona, ni en Tánger. Esto se convirtió en un resultado en sí mismo, ya que los eventos se convocaron de forma puntual y la temática no tuvo ninguna continuidad a través de más eventos ni conferencias. Así, las preguntas sobre posibles eventos y actividades también formaron parte de la guía de entrevista para los líderes musulmanes y miembros de asociaciones con el fin de conocer por qué no se estaban realizando eventos similares. 

## RESULTADOS

### Islam y ciencia

Para poder analizar la relación entre ciencia y religión desarrollando lo que Lamont define como “*boundary work*”^9^^,^^10^, pregunté directamente a las personas entrevistadas sobre su forma de definir y entender religión y ciencia. Como abordaré en las siguientes líneas, no surgieron diferencias relevantes en las dos ciudades y los entrevistados defendieron enfoques similares. Así, la característica más común fue la similitud en su insistencia en defender una relación complementaria entre el islam y la ciencia. Para mostrar este tipo de relación surgieron dos aspectos principales: 1) el Corán como fuente de conocimiento; y 2) el conocimiento científico que refuerza la fe y la creencia.

#### El Corán como fuente de conocimiento

La mayoría de los entrevistados argumentaron que la relación entre religión y ciencia se puede entender como complementaria, haciendo referencia al Corán como la principal fuente de conocimiento científico. Su principal argumento es que en el Corán ya hay temas científicos que la ciencia ha demostrado. Por ejemplo, un farmacéutico musulmán nacido en Barcelona argumentó lo siguiente:

*Yo puedo explicar un poco la relación que veo entre el islam y la ciencia como musulmán, porque en el propio Corán, en el libro que seguimos y por el cual nos guiamos para practicar nuestra religión, ya tiene fundamentos científicos y tiene muchas escrituras que hablan de ciencia, desde cómo se genera un feto en el embrión de la madre, hasta temas astrológicos, el universo, el sol, los planetas y las estrellas, incluso los animales, habla sobre cómo los animales se relacionan entre sí, el tema de los genes, que somos muy similares al cerdo, por ejemplo, a nivel genético, el Corán ya habla de esto y hay muchas cosas que ya están científicamente demostradas que el Corán ya te lo explica*. (EB07)

Como describe este farmacéutico, el Corán incluye información sobre una amplia gama de temas que la ciencia demostró después de que se reveló el libro sagrado. De manera similar, una médica también relacionó los hallazgos científicos con las descripciones islámicas para demostrar que la ciencia y la religión no son áreas contradictorias, sino complementarias:

*...la mayoría de las cosas que la ciencia está descubriendo ahora o ha descubierto recientemente, ya están descritas en el islam. Puede ser nuevo al principio, en el sentido de entender lo que dice el Corán o lo que nuestro profeta nos ha enseñado, pero al final termina llegando a la misma conclusión, tarde o temprano, pero no son cosas contradictorias. Además, no son cosas que se contradigan entre sí, se combinan*. (EB03)

En Tánger, se dio la misma respuesta por parte de un médico que también definió la relación entre el islam y la ciencia estableciendo una diferencia entre el islam y otras religiones como el budismo:

*¿Qué tiene que ver la religión con la ciencia? Por ejemplo, el budismo no tiene mucho que ver con la ciencia, trata todo lo espiritual. Te doy el punto de vista del islam, si trata la ciencia, no directamente, no podemos encontrar la diabetes en el Corán, pero encontraremos versos que incitan a las personas a trabajar y a aprender, es como un motor espiritual que te empuja a trabajar, aquellos que no trabajan, que no buscan, su islam es débil, está en el Corán, es el hadiz. Tienes que trabajar, tienes que buscar, eso es básicamente todo. De vez en cuando en el Corán encontramos versos que no son estudios, pero es el resultado de los estudios*. (ET03)

Así, a través de estas citas podemos ver cómo los informantes no establecen límites simbólicos excluyentes entre “ciencia” y “religión” sino que recurren al Corán como la principal evidencia de la relación entre los dos ámbitos y la ausencia de conflicto^4^^,^^5^^,^^6^. Con el fin de ahondar en posibles tensiones en esta relación, les pregunté a los informantes sobre posibles teorías científicas que pudieran contradecir a las ideas religiosas. Esta pregunta era relevante especialmente en los informantes de Barcelona, ya que se encuentran en un contexto más minoritario. En la mayoría de las ocasiones, respondieron a esta pregunta haciendo mención a la teoría de la evolución que se suele enseñar en los colegios. Sin embargo, ninguno de ellos le dio importancia y lo consideraron como una teoría más que se estudia, una percepción que coincide con otro estudio que reveló cómo la evolución no es un tema relevante ni conflictivo entre musulmanes^8^^,^^26^. 

#### El conocimiento científico que refuerza la fe y la creencia

El segundo aspecto que emergió como consecuencia de la afirmación de una relación complementaria entre el islam y la ciencia basada en el conocimiento científico es el impacto en la identificación religiosa. En las entrevistas con profesionales de la salud, estaba particularmente interesada en saber si sus decisiones de estudiar medicina, enfermería o farmacia tuvieron un impacto en modificar su aproximación a las definiciones de ciencia y religión. Estudios anteriores ya habían señalado que los musulmanes en contextos minoritarios, como Gran Bretaña, pueden mostrar una preferencia por las disciplinas relacionadas con la ciencia como parte de su ambición de movilidad social y económica^8^. En este caso, al reflexionar sobre la relación entre ciencia y religión, algunos participantes afirmaron volverse “más religiosos” y tener “más fe”, después de estudiar ciencias y conocer más sobre el cuerpo y el mundo. Esto fue especialmente evidente en el caso de una médica nacida en Barcelona:

*No encuentro ninguna incompatibilidad entre la religión y la ciencia, todo lo contrario, muchas veces en la ciencia o estudiando he tenido más fe porque, por ejemplo, en el segundo año de la carrera estábamos estudiando fisiología, embarazo y era una clase donde estudiamos el calostro. El calostro es la primera leche materna que sale, que son los primeros días y es diferente del resto, porque en lugar de leche es como un líquido amarillento que tiene más anticuerpos y proteínas, que es lo que el recién nacido necesita y tiene algunos nutrientes que son los que el recién nacido puede digerir según la madurez de su tracto digestivo. Pensé “es que es tan perfecto, es justo la sustancia que está saliendo lo que el recién nacido necesita” y esto está evolucionando. Mucha gente dice “Madre Naturaleza”, pero lo que es Madre Naturaleza para ti, que es sabia, para mí es Dios detrás de ella, es como una entidad que hace todo tan perfecto, así que no es incompatible*. (EB02)

En Tánger, una embrióloga que trabaja en un centro de fertilidad declaró algo similar, pero en referencia al conocimiento proporcionado en el Corán. También mencionó ser “más religiosa” después de estudiar embriología y trabajar en el centro:

*...hay cosas en la ciencia de hoy en día que prueban algunas cosas que fueron dichas hace años en el Corán por Dios y todo. Y se demuestran hoy. Sí. Así que eso es realmente lo que te hace más religioso. Y puedo decir que es algo personal. Yo soy como, he sido más religiosa, especialmente cuando descubrí este campo*. (ET02)

Estas citas evidencian como el trabajo de definición de límites o “*boundary work*” entre ciencia y religión puede tener un impacto claro en su subjetividad como musulmanas. Así, el conocimiento científico refuerza lo religioso y viceversa, complejizando la relación entre ciencia y religión y confirmando su identificación religiosa. 

Otro aspecto relevante de esta definición de complementariedad es lo relativo al papel importante de la religión en el éxito de los tratamientos. Reconocen que la religión podría complementar el papel científico, especialmente durante los tratamientos. Entonces, según explicó un embriólogo que trabaja en una clínica de fertilidad:

*Las personas en el islam suelen hacer los tratamientos que son necesarios, pero aparte de eso, suelen hacer muchas súplicas a Dios. Incluso hay un dicho, más o menos, como cuando quieres algo, en el islam cuando quieres algo de corazón, haces una súplica a Dios, pero si, por ejemplo, quieres estudiar Medicina, tienes que pedirle a Dios, pero tienes que pasar un examen, es ambas cosas, no vale con “voy a pedirle a Dios” y ya está, tienes que hacerlo para que Dios te ayude, si solo le pides, pero no haces nada para que Dios te ayude, no te ayudará, si estudias para el examen y le pides a Dios por ello, entonces lo harás mejor, ambas cosas son necesarias. Así que, en un tratamiento, la gente va al hospital o al tratamiento que se debe hacer, pero aparte de la fe de que Dios los ayudará, ambas cosas*. (ET05)

Esta importancia de pedir a Dios es particularmente evidente en el caso de los tratamientos de reproducción asistida. Algunos profesionales de la salud y concretamente ginecólogas reconocen el papel del rezo en los tratamientos médicos y en creer en el éxito de los tratamientos médicos. Además, tal y como veremos en el próximo apartado, cuando se tratan avances científicos como los de las técnicas de reproducción asistida, la relación entre ciencia y religión se complejiza todavía más. 

#### El islam y los tratamientos de reproducción asistida

Entre los diversos temas que surgieron al preguntar sobre los tratamientos de reproducción asistida, cabe destacar las siguientes cuestiones: 1) conocimiento sobre el islam y técnicas de reproducción asistida; 2) las donaciones y el parentesco; y 3) las alternativas al uso de donantes. Además, entre estos temas surgieron algunas diferencias interesantes entre las entrevistas realizadas en Barcelona y Tánger.

#### Conocimiento sobre el islam y las técnicas de reproducción asistida: entre el desconocimiento y la discreción

El primer elemento para destacar es que algunos informantes no sabían mucho sobre los tratamientos que están permitidos por la ley islámica. Esto sucedió especialmente en Barcelona, ​​ya que la mayoría de las personas participantes, a pesar de ser profesionales de la salud o líderes de asociaciones, no conocían los detalles sobre este tema. Esta ignorancia puede explicarse por el problema de tener menos acceso a la información en un contexto minoritario como el de Barcelona. Los profesionales de la salud -que eran enfermeros, farmacéuticos o médicos generales- reconocieron que no aprendieron sobre la perspectiva islámica y la salud en la universidad, por lo que necesitan buscar fuentes islámicas para aprender sobre esto. Entre los responsables de asociaciones islámicas, tampoco había mucho conocimiento profundo sobre el tema. En esa línea, es interesante destacar que una de las entrevistadas era la líder de un grupo de mujeres de una asociación chiita en Barcelona. Cuando le pregunté sobre las técnicas de reproducción asistida, me reconoció que no conocía las especificidades de la perspectiva chiita y los tratamientos aceptados. Concretamente, su respuesta fue:

*Bueno, la verdad es que siempre quiero saber si está permitido o no según el islam chiita, pero no lo sé. Sé que el aborto, sí, tienden a abortar y cosas así, pero creo que la fertilidad también tiende a hacerlo.* [….] *En Pakistán, muchas personas suelen ir a los médicos en busca de medicamentos para tener hijos o tratamientos, por eso te digo, no está prohibido*. (EB06)

Cuando le comenté que el islam chiita permitía más tratamientos que el islam suní, mencionó rápidamente al islam chiita como una religión “más abierta”, estableciendo una frontera entre las dos ramas principales del islam:

*El islam chiita es más abierto que el sunismo, por eso nos dicen que no somos musulmanes, también tenemos tatuajes, hay muchos chiitas que tienen tatuajes, especialmente con el nombre del Imam Ali y cosas así*. (EB06)

En cambio, en Tánger fue más fácil hablar sobre los tratamientos de infertilidad permitidos en el islam. A pesar de la mayor facilidad para acceder a ginecólogos y embriólogos musulmanes, también pude hablar del tema con médicos generales. Siendo un país en el que la mayoría de los musulmanes practican el islam suní, el discurso general era que la donación de terceros no estaba permitida. Esto podría explicarse fácilmente por la existencia de un contexto legal religioso que enmarca los tratamientos de fertilidad y que permite menos dudas entre la población marroquí. Sin embargo, a pesar de ser un tema más conocido, muchos informantes afirmaron que era un tema difícil de tratar en público. Aunque existen clínicas de fertilidad en el país, su trabajo destaca por ser muy discreto. Por ejemplo, una embrióloga reconoció que intentaban hacer pocas visitas para hacer coincidir poca gente un mismo día y que no se encontraran en la sala de espera. Además, reconocía que para muchas parejas no era común decir que habían tenido un hijo por reproducción asistida:

*Entonces, incluso si las personas tienen éxito en el tratamiento de FIV* [fecundación in vitro]*, tienen un bebé sano y todo, no pueden hablar de eso. No pueden socialmente, no es realmente aceptable que la pareja diga: “Por cierto, tuvimos un tratamiento de FIV y así fue como tuvimos a nuestro bebé”. La mayoría de las veces simplemente no quieren compartir esta información. Es por eso por lo que, como los centros de FIV en Marruecos, la gente no hace la promoción del centro. Sabes, no pueden decir, “oh, tuve una experiencia muy hermosa aquí. Puedes ir allí si tienes problemas”. Y la gente, cuando viene aquí, tratamos de manejar nuestros horarios. Así que no puede venir mucha gente*. (ET02)

El poco conocimiento sobre las especificidades del tema y la discreción con la que se suele tratar puede explicar también que no haya podido observar eventos ni charlas sobre reproducción asistida. En Barcelona, una mezquita organizó dos eventos sobre ciencia e islam, pero centró su explicación en el ámbito del covid-19 y las restricciones. Aunque muchos informantes reconocieron querer saber más sobre las especificidades del islam en referencia a las técnicas de reproducción asistida, ninguna asociación ni centro religioso se ha planteado tratar este tema en el ámbito público. Este aspecto contrasta con lo que evidencian otras investigaciones sobre el papel activo de actores religiosos en el catolicismo y su movilización activa de discursos científicos legitimadores de la Iglesia Católica en distintos contextos^27^^,^^28^.

#### Las donaciones y el parentesco

El segundo elemento clave a destacar es el relativo a las donaciones y los debates bioéticos sobre el parentesco. A pesar de las dificultades de tratar este tema en profundidad, la mayoría de los informantes que conocían sobre las técnicas de reproducción asistida permitidas en el islam hacían referencia a la imposibilidad de hacer uso de donantes anónimos de terceros. Así, insistían en que la fertilización asistida es posible únicamente con el uso de óvulos y espermatozoides de dos personas casadas. Tal y como detallaba un imam de Barcelona:

*…utilizan máquinas y el óvulo de la madre, el islam lo permite, pero lo que no permite el islam es que el óvulo de la mujer sea fertilizado por espermatozoides que no sean de su marido, ahí radica el problema, solo tiene que ser el óvulo de su esposa y el esperma de su esposo, en ese caso pueden intentar fertilizar, pueden hacer lo que quieran, pueden hacer todo lo posible para que puedan tener hijos y pueden resolver su problema. Pero, por supuesto, eso no puede causar que se incluya a otra mujer o a otro hombre, no está permitido*. (EB09)

El tema del matrimonio emergió como clave en todas las conversaciones sobre las posibilidades de hacer uso de técnicas de reproducción dentro de los principios islámicos. Mientras que en Barcelona los informantes no me pudieron explicar estrategias de la comunidad musulmana para resolver posibles dudas sobre las técnicas de reproducción asistida y el parentesco, en Tánger, los posibles debates bioéticos sobre la genética y el parentesco se resuelven con una estricta regulación que está presente en todo el país. La primera forma de controlar que los tratamientos estén permitidos religiosamente es pidiendo un certificado de matrimonio a las parejas que quieren hacer el tratamiento. Tal y como explicaba una embrióloga que trabaja en una clínica de fertilidad:

*...solo las parejas casadas pueden recibir tratamiento de FIV. Incluso cuando tenemos en su carpeta sus análisis y todo, debemos poner su certificado de matrimonio. Si la pareja se niega a darnos esto, podemos negarnos a darles el tratamiento*. (ET02)

La preocupación por el parentesco y la genética también lleva a que las clínicas de fertilidad efectúen protocolos estrictos para asegurar que no se producen mezclas con las muestras genéricas. Además, en las clínicas también tratan de tener muy pocos casos, para asegurarse de que no haya errores con los óvulos:

*Oh, sí, tenemos que verificarlo dos veces y no tenemos cómo hacer dos recolecciones de óvulos en un día. A veces programamos una por día. Vale, cuando podemos, porque no es como en Europa o Estados Unidos. No tenemos muchos casos de FIV. Es por eso que podemos programar una función por día. Así que hay un sistema de calidad que podemos aplicar.* (ET02)

Otra consecuencia de la importancia del matrimonio y el parentesco en las técnicas de reproducción asistida es que deja fuera la posibilidad de realizar tratamientos de fertilidad para mujeres solteras. Así lo reconoció una responsable de una asociación de mujeres en Tánger:

*Creo que es un paso demasiado lejano hablar de tratamiento de fertilidad para madres solteras. Es tan, tan vergonzoso. Actualmente, no estoy segura de que una mujer esté dispuesta a ser una madre soltera y decidir tener tratamiento para hacerlo*. (ET08)

Finalmente, cabe destacar que la preocupación por el uso correcto de muestras genéticas de personas casadas contrastaba con el poco debate sobre el uso de muestras genéticas en un laboratorio. Este tema no surgió en las entrevistas seguramente porque en el islam no se considera la vida hasta dentro de 40-120 días después de la fertilización y el uso de embriones no se considera una muerte porque no se considera todavía un ser humano^3^. Mientras que este es uno de los temas claves en el catolicismo analizado profundamente por varias investigadoras, la bioética islámica parece no plantear debate en torno al descarte de embriones, la criopreservación y el estatus de los embriones no implantados^29^^,^^30^. 

#### Las alternativas al uso de donantes

La tercera cuestión clave es la relativa a las alternativas que las parejas musulmanas se pueden plantear para poder ser padres. Uno de los retos de la bioética islámica es la tensión entre la existencia de una ética universal y una ética que se acomode a los principios religiosos específicos del islam^2^. En los tratamientos de reproducción asistida, la diversidad religiosa existente en Europa podría plantear un reto: la posibilidad de que haya países que permitan unos avances tecnológicos y que los miembros religiosos de ese país tengan que gestionar y decidir si siguen estos tratamientos según sus principios o no. Es decir, a partir de la diversidad religiosa existente en ciudades como Barcelona, se podría generar la ausencia de una homogeneidad en la percepción de los debates bioéticos en torno el parentesco y la genética. 

Sin embargo, según las personas entrevistadas en Barcelona, es difícil afirmar que personas musulmanas se cuestionen alternativas que vayan en contra de los principios islámicos. Una farmacéutica, por ejemplo, destacó que, aunque haya musulmanes que hagan cosas *haram* o prohibidas por la religión, el uso de donantes en técnicas de reproducción asistida es un pecado que tendría un impacto muy directo en sus vidas:

*A ver, cosas no permitidas se hacen muchas, piensas una más, pero esto es un tema que estás trayendo a una persona al mundo y una persona que vas a tener toda la vida contigo, no es una cosa momentánea, que tú la haces y al día siguiente te olvidas, es algo que estás haciendo siempre, entonces pienso que la consecuencia de hacer este pecado es mucho más, tiene mucho más peso porque es muy duradera, es toda la vida, que no es algo que haces que no está permitido*. (EB07)

En Tánger, el marco legal y religioso existente deja poco espacio para renegociar la posibilidad de usar otras técnicas no permitidas por el islam, más allá de lo que otras autoras han destacado como “turismo reproductivo”. Allí una ginecóloga reconoció que conocía a parejas que viajaban a España o Francia para recibir tratamiento de fertilidad con donantes, pero no quiso dar detalles al respecto aludiendo al secreto profesional. Mientras que fue difícil obtener datos sobre posibles viajes a países no-musulmanes para poder renegociar los tratamientos permitidos, muchos informantes mencionaron viajes con el recorrido inverso. Es decir, viajes de parejas que viven en Europa y viajan a Marruecos, generalmente, para poder tener tratamientos de reproducción asistida más baratos. Así lo explicaba una trabajadora de una clínica:

*A veces tenemos parejas de Europa porque aquí es más barato para ellos y a veces solo quieren hacer el tratamiento de FIV aquí. Así que, por ejemplo, en Marruecos, porque es como turismo más tratamiento de FIV, es algo bueno porque no están todo el tiempo estresados. Es realmente difícil para, por ejemplo, una mujer que trabaja y hace el tratamiento de FIV, ya sabes, hay hormonas y cambios de humor y todo. Así que es realmente bueno para las parejas venir de países extranjeros y tener vacaciones más tratamiento de FIV*. (ET02)

Mientras que algunas perspectivas cuestionen el uso del concepto “turismo”, por la imposibilidad de captar la dureza y las dificultades por las que pueden pasar las parejas durante los tratamientos^19^, destaca el uso que hace la embrióloga de la palabra “vacaciones” para destacar como algo positivo la posibilidad de viajar y únicamente dedicarse al tratamiento durante la estancia. Además, y como muestra esta cita, es el tema económico y no la cuestión religiosa la que parece explicar el “turismo reproductivo”. Todos los profesionales en Tánger insistieron en que en Marruecos es más barato que en el resto de Europa, pero no le dieron importancia ni a temas religiosos ni sanitario. Así, esto muestra que también es relevante abordar cómo los problemas sociales y económicos se cruzan con los dilemas religiosos, ya que estos tratamientos suelen ser costosos^22^.

Finalmente, otra alternativa que emergió en las conversaciones sobre las dificultades de renegociar los principios islámicos y ante posibles fracasos de tratamientos de fertilidad es la de la adopción. Así, si las técnicas de reproducción asistida no funcionan con el material genético del matrimonio, la adopción podría ser la única alternativa para el matrimonio. Sin embargo, según comentaron varios informantes, lejos de resolver los debates sobre genética y parentesco, la adopción reabre esta cuestión ante la duda sobre la posibilidad de tratar a un hijo adoptado como hijo. Un *sheik* de Tánger explicó claramente cómo en el islam está permitido acoger, pero no adoptar, precisamente por unos motivos muy parecidos a los de la imposibilidad de usar donantes anónimos en las técnicas de reproducción asistida:

*…la adopción no está permitida en el islam, pero sí lo que es la acogida, lo que es cuidar a un huérfano al que no le vas a dar tus apellidos, porque no está permitido, él siempre tiene que saber que no son sus padres…* […] *Lo que es acoger a un niño o niña que es huérfano, que no se sabe quién son sus padres y cuidarle y criarlos como hijos es una de las mejores obras que una persona puede hacer, lo que no está permitido es mentirles y decirles que “soy tu padre” y darles tus apellidos o ponerlos a la misma altura que tus hijos, no*. (ET01)

A pesar de su explicación más clara, otros informantes mencionaron la adopción como una alternativa a la posible infertilidad sin cuestionar si era algo permitido en el islam ni plantear dilemas bioéticos. Otra vez más, el desconocimiento sobre las particularidades de la bioética islámica marca la negociación entre islam y ciencia, ofreciendo un panorama complejo para las parejas que tienen que tomar decisiones antes posibles problemas de infertilidad. 

## CONCLUSIONES

Tres conclusiones principales emergen de este artículo, que pueden servir para futuras investigaciones más extensas y detalladas sobre los debates bioéticos en el islam y la ciencia y que, a su vez, pueden contribuir a los debates existentes sobre bioética, religión y salud^12^^,^^27^^,^^31^. La primera conclusión es relativa al caso específico del islam para abordar la relación entre ciencia y religión. A pesar de las investigaciones ya desarrolladas sobre la compleja relación entre religión y ciencia, este caso de estudio muestra la importancia de abordar el caso del islam desde la perspectiva de las propias personas musulmanas. La investigación muestra cómo las referencias científicas presentes en el Corán sirven para que las propias personas musulmanas puedan definir una relación de complementariedad entre religión y ciencia. Además, esta definición tiene un papel relevante en la redefinición de la identificación de las personas religiosas, sobre todo de aquellas implicadas en el campo científico. Tal y como afirma Gülker^6^, este artículo muestra la relevancia de abordar la relación entre ciencia y religión de forma empírica y a partir de las propias definiciones que hacen las personas implicadas. 

Segundo, el artículo muestra cómo la relación entre ciencia y religión se vuelve más compleja cuando se tratan temas como la reproducción asistida, pero sin necesidad de recurrir a la tesis del conflicto. Es decir, la discusión sobre técnicas de reproducción asistida revela cómo hay avances científicos no permitidos por algunos principios islámicos y cómo los discursos bioéticos se vuelven presentes a la hora de analizar los avances científicos y tecnológicos a partir de una perspectiva religiosa^3^. Sin embargo, esto no hace cuestionar la relación de complementariedad entre religión y ciencia, sino que precisamente demuestra que la religión musulmana es un factor relevante a la hora de tomar decisiones sobre tratamientos^32^. A pesar de la dificultad de tratar estos temas y encontrar personas que conozcan los detalles sobre las técnicas de reproducción asistida en el islam, la bioética islámica permite poner los límites ante los avances científicos. Así, tal y como hemos discutido, las clínicas de fertilidad en Tánger establecen requerimientos legales además de incluir estrategias para asegurar que las técnicas sean aceptadas y mejorar la experiencia de los pacientes: incluir certificados de matrimonio en expedientes, seguir protocolos estrictos para evitar mezclas de material genético y establecer pocas visitas al día. La investigación también ha mostrado cómo es un tema ausente en la esfera pública, y tampoco parece que haya agentes políticos ni líderes de asociaciones que aborden esta cuestión. 

Tercero, el artículo también ha mostrado cómo el contexto local es importante a pesar de partir de las mismas concepciones sobre la relación entre ciencia y religión y sobre los debates bioéticos islámicos. El marco legal en Tánger, una ciudad de un país en el que se practica el islam sunní, hace que las posibilidades de renegociación de técnicas de reproducción asistida sean más difíciles de abordar. Sin embargo, el contexto de minoría religiosa en el que se encuentran los musulmanes y musulmanas que viven en Barcelona muestra la potencialidad para abordar este tema en especial de las parejas infértiles o con dificultades para tener hijos e hijas. La conexión transnacional solo ha emergido por temas económicos en los posibles viajes a Marruecos para acceder a tratamientos más baratos. A pesar de las dificultades metodológicas y éticas que podrían aparecer, una investigación centrada en parejas que hayan tenido que tomar decisiones sobre tratamientos de reproducción asistida podría abordar de forma más detallada cuestiones como posibles estrategias de renegociación de uso de donantes, así como las diferencias entre hombres y mujeres en la toma de decisiones sobre tratamientos de reproducción asistida.
